# The Role of Kisspeptin in the Pathogenesis of Pregnancy Complications: A Narrative Review

**DOI:** 10.3390/ijms23126611

**Published:** 2022-06-14

**Authors:** Magdalena Szydełko-Gorzkowicz, Elżbieta Poniedziałek-Czajkowska, Radzisław Mierzyński, Maciej Sotowski, Bożena Leszczyńska-Gorzelak

**Affiliations:** Department of Obstetrics and Perinatology, Medical University of Lublin, Jaczewskiego 8, 20-090 Lublin, Poland; elzbieta.poniedzialek@umlub.pl (E.P.-C.); radekm1969@gmail.com (R.M.); maciek13011@wp.pl (M.S.); b.leszczynska@umlub.pl (B.L.-G.)

**Keywords:** kisspeptin, kisspeptin-1 receptor, gestational diabetes mellitus, preeclampsia, implantation, placenta, pregnancy complications

## Abstract

Kisspeptins are the family of neuropeptide products of the *KISS-1* gene that exert the biological action by binding with the G-protein coupled receptor 54 (GPR54), also known as the KISS-1 receptor. The kisspeptin level dramatically increases during pregnancy, and the placenta is supposed to be its primary source. The role of kisspeptin has already been widely studied in hypogonadotropic hypogonadism, fertility, puberty disorders, and insulin resistance-related conditions, including type 2 diabetes mellitus, polycystic ovary syndrome, and obesity. Gestational diabetes mellitus (GDM), preeclampsia (PE), preterm birth, fetal growth restriction (FGR), or spontaneous abortion affected 2 to 20% of pregnancies worldwide. Their occurrence is associated with numerous short and long-term consequences for mothers and newborns; hence, novel, non-invasive predictors of their development are intensively investigated. The study aims to present a comprehensive review emphasizing the role of kisspeptin in the most common pregnancy-related disorders and neonatal outcomes. The decreased level of kisspeptin is observed in women with GDM, FGR, and a high risk of spontaneous abortion. Nevertheless, there are still many inconsistencies in kisspeptin concentration in pregnancies with preterm birth or PE. Further research is needed to determine the usefulness of kisspeptin as an early marker of gestational and neonatal complications.

## 1. Introduction

Pregnancy is a unique physiological condition associated with several anatomical, biochemical, and metabolic changes to provide adequate nurture and accommodate the growing fetus [1]. The proper implantation and invasion of syncytiotrophoblast cells into the uterine spiral arteries are the crucial stages responsible for the successful course of pregnancy and neonatal implications [2]. The proper placentation ensures the development of optimal placental circulation enabling the supply of nutrients and oxygen for the developing fetus and the removal of fetal metabolic products to maternal circulation [3]. Moreover, the placenta is considered a multifunctional organ that produces and releases hormones to the maternal and fetal compartments [1]. Impairment in placental function may lead to the development of pregnancy-related disorders, including spontaneous abortion, preeclampsia (PE), fetal growth restriction (FGR), gestational diabetes mellitus (GDM), or preterm birth [4,5,6,7]. The prevalence of these disorders ranges from 2 to 20%, depending mainly on the quality of medical care and socioeconomic conditions [8]. Despite the considerable advances in their prevention, diagnostics, and monitoring, there are still many ambiguities about their pathogenesis. Many of these gestational complications have been previously reported to be associated with the family of kisspeptin and its receptor [9,10,11].

Kisspeptins are the peptide products of the *KISS-1* gene, which was first identified in 1996 in Hershey, Pennsylvania (USA) as a human metastasis suppressor gene [12]. Kisspeptins exert biological action by binding with the G-protein coupled receptor 54 (GPR54), also known as the KISS-1 receptor (KISS-1R) [13]. Kisspeptins and KISS-1R participate in different biological processes due to their expression in various tissues, including the pancreas, adipose tissue, liver, small intestine, brain, hypothalamus, adrenal glands, testes, as well as smooth muscles cells of the aorta, coronary artery and umbilical vein [14,15,16]. It is widely known that kisspeptin plays an essential role in the central regulation of pubertal onset and human reproduction. The study conducted by Messager et al. revealed that kisspeptin intracerebroventricular administration leads to elevated gonadotrophin-releasing hormone (GnRH) in cerebrospinal fluid. Moreover, this experimental study on an animal model has proved that GPR54^−^/^−^ mice could not respond with the increase in GnRH secretion to the supply of kisspeptin [17]. Further studies confirmed the hypothesis that kisspeptin could regulate the hypothalamus-pituitary-gonad (HPG) axis, and the mutation of KISS-1R is responsible for the development of idiopathic hypogonadotropic hypogonadism [18]. 

The discovery of kisspeptin/GPR54 expression in human and mouse pancreatic β-islet cells strongly indicated its potential effect on glucose homeostasis [19]. Numerous in vitro and in vivo studies have revealed that this family of peptides can stimulate insulin secretion in a glucose-dependent manner [20,21,22]. A study conducted by Tolson et al. has shown that female mice lacking the kisspeptin gene presented higher body weight, increased leptin concentration, and thus a higher level of insulin resistance. Interestingly, such results were not obtained in male individuals, suggesting that the effect of kisspeptin in the regulation of glucose homeostasis is sex-specific [23]. Furthermore, the expression of this peptide in the hypothalamic arcuate nucleus controls the appetite, food intake, and energy balance [24]. Data suggests that kisspeptin exhibits the properties of anorexigenic hormones such as adiponectin and glucogenic-like peptide and inversely correlates with orexigenic hormones’ concentrations [25,26,27]. These results oppose those obtained by Orlando et al., demonstrating that kisspeptin may decrease brain-derived neurotrophic factors and increase the level of neuropeptide Y, which stimulates glucose uptake [25].

In placental tissues, KISS-1 mRNA and kisspeptins have been previously detected in syncytiotrophoblast and, to a lesser degree, in cytotrophoblast, whereas KISS-1R has been found to be expressed in syncytiotrophoblast, villous and invasive extravillous trophoblast [28]. In Vitro experimental studies have shown decreased expression of kisspeptin/GPR54 in women with recurrent spontaneous abortion, thus suggesting that kisspeptin is engaged in the embryo implantation process [29]. Furthermore, the role of kisspeptin in developing diseases from the spectrum of impaired placentation, including PE and FGR, has been widely studied [11].

However, in the available literature, there is still a lack of comprehensive review involving the role of KISS-1R, and its ligands in pregnancy-related complications, including GDM, PE, FGR, preterm birth, and spontaneous abortion. Moreover, based on the latest research on the role of the placenta in fetal metabolic programming, changes in kisspeptin levels in the development of neonatal outcomes remain a crucial issue [30].

## 2. Scope and Methodology

The review aims to systematize the available information on the involvement of kisspeptin and its derivatives in the most common pathological conditions related to pregnancy. A list of analyzed disorders was restricted to relatively well-studied complications, including GDM, PE, FGR, spontaneous abortion, and preterm birth. Furthermore, the role of kisspeptin in neonatal outcomes was discussed. To establish a list of relevant references, PubMed and MEDLINE databases were searched from the first records until January 2022 using MeSH Terms such as: ‘kisspeptin’, ‘kisspeptin 1 receptor’, ‘gestational diabetes mellitus’, ‘preeclampsia’, ‘fetal growth restriction’, ‘implantation’, ‘placenta’, and ‘pregnancy complications’ as keywords. The references included in these selected publications were also analyzed to find additional essential publications. Only articles in English were considered. 

## 3. The *KISS-1* Gene, Kisspeptin and Its Signaling

The *KISS-1* gene in humans is located on the long (q) arm of chromosome 1 at q32 and contains four exons. Exons 3 and 4 encode an intermediate prepropeptide of the 145 amino acids (kisspeptin-145), an unstable and biologically inactive precursor [31]. Then, kisspeptin-145 undergoes post-translational proteolytic processes, which lead to the production of four shorter active peptides, including kisspeptin-54, also known as metastin (54 amino acids), kisspeptin-14 (14 amino acids), kisspeptin-13 (13 amino acids), and kisspeptin-10 (10 amino-acids) [32,33]. Kisspeptin-54 is considered the straight conversion product of kisspeptin-145, while the other three isoforms probably originate from the degradation of kisspeptin-54. Moreover, these peptides can activate their shared receptor with the same affinity and efficiency due to the presence of C-terminal regions, which contains an Arg-Phe-NH2 signal motif [33,34]. This peptide family has been called ‘kisspeptins’ due to their structural, functional, and origin-based similarities.

KISS-1R, also known as GPR54, AXOR12, CPPB1, hOT7T175, or HH8, belongs to class A (rhodopsin-like) G protein-coupled receptor (GPCR) family [32,33,35,36,37]. The human *KISS-1R* gene consists of five exons and encodes a peptide chain of 398 amino acids. This transmembrane receptor reveals homologies of the sequence with galanin (about 45% sequence identity) and somatostatin receptors, which explains why KISS-1R was previously considered an orphan receptor before it was recognized as a putative receptor of kisspeptin [32,35,38]. 

Kisspeptin binding with its receptor leads to the activation of phospholipase C (PLC) by phosphorylated Gq/11 protein, which triggers cascade conversions of phospatidylinoinositol-4,5-diphosphate (PIP_2_) into diacylglycerol (DAG) and inositol-(1,4,5)-triphosphate (IP_3_). In the next step of this signaling pathway, the subsequent mobilization and secretion of Ca^2+^ to the cytoplasmic surface activate protein kinase C (PKC), which phosphorylates mitogen-activated protein kinases (MAPK) such as the extracellular signal-regulated kinase (ERK) and p38 [33,39,40]. 

However, kisspeptin-induced phosphorylation of ERK1/2 but not p38 seems to be the crucial signaling pathway in some types of cells [33,39,41]. The results of the experimental studies have revealed that kisspeptin may promote β-arrestin 1 and β-arrestin 2 migration to the plasma membrane and lead to the elevated intracellular level of phosphorylated ERK1/2 as a consequence [42,43]. 

Recent in vitro studies have indicated that kisspeptin-10 exerts an anti-angiogenic effect by inhibiting the migration, invasion, and tube formation of human umbilical vein endothelial cells by suppressing the expression of vascular endothelial growth factor (VEGF) [44,45]. This hypothesis has been confirmed by Francis et al., who have demonstrated that the administration of kisspeptin-10 to human trophoblast cells decreased their invasion and angiogenic abilities by reducing the expression of matrix metalloproteinase (MMP) 1, 2, 3, 7, 9, 10, 14, and VEGF, and increasing the expression of tissue inhibitors of metalloproteinases (TIMPs) 1 and 3 [46]. 

**Figure 1:**
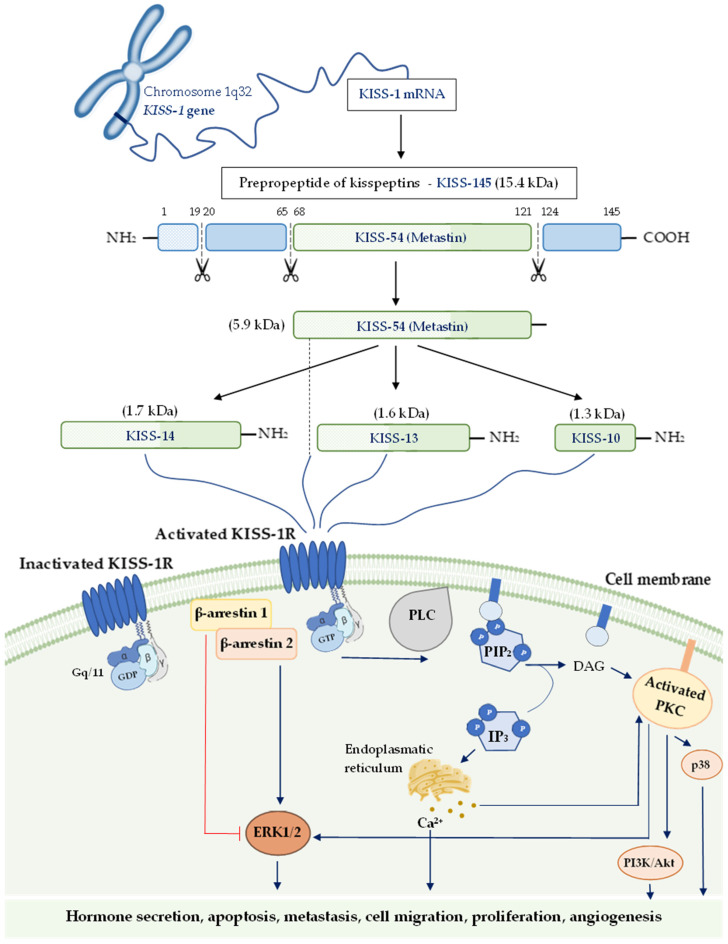
The process of kisspeptin isoforms formation and their putative signaling pathways. The activation pathways are shown as blue lines and inactivation ones in red. Abbreviations: *KISS-1* gene—kisspeptin gene; KISS-145—kisspeptin-145; KISS-54—kisspeptin-54; KISS-14—kisspeptin-14 KISS-13—kisspeptin-13; KISS-10—kisspeptin-10; KISS-1R—KISS-1 receptor; PLC—phospholipase C; PIP_2_—phospatidylinoinositol-4,5-diphosphate; DAG—diacylglycerol; IP_3_—inositol-(1,4,5)-triphosphate; PKC—protein kinase C; PI3K/Akt—phosphatidylinositol 3-kinase/Akt; ERK—extracellular signal-regulated kinase.

Although kisspeptin and its receptor were discovered many years ago, the data on the factors regulating its activity is still limited. It is suggested that binding KISS-1R with the ligand in the placenta may decrease their expression in syncytiotrophoblast, as in thyrotropin-releasing hormone [47]. The latest experimental research indicates that transforming growth factor β-1 (TGF β-1) upregulates kisspeptin expression in human extravillous cytotrophoblast cells by inhibiting the ERK1/2 signaling pathway and, in this way, it leads to decreased trophoblast cell invasion [48]. The process of kisspeptin isoforms formation, as well as its various signaling pathways, are presented in Figure 1.

## 4. Kisspeptin Level throughout Pregnancy

The first evidence regarding changes in the concentration of kisspeptin in human plasma was delivered by Horikoshi et al. in 2003 [49]. This study has revealed that the plasma level of kisspeptin-54 did not differ between men and non-pregnant women. However, the mean concentration of metastin increases markedly throughout pregnancy, with a 900-fold elevation in the first trimester (1230 ± 346 fmol/mL) and over a 7000-fold growth in the third trimester (9590 ± 1640 fmol/mL) as compared to non-pregnant women (1.31 ± 0.37 fmol/mL). The significant decrease in the kisspeptin level within 5 days post-partum (7.63 ± 1.33 fmol/mL) and the confirmed kisspeptin expression in the placenta have suggested that the placenta remains the main source of kisspeptin during pregnancy [28,49]. Moreover, no differences between metastin concentrations in umbilical arterial and venous blood have been observed [49]. These results are consistent with those obtained by Abbara et al., who have shown that gestational age was positively associated with the plasma level of kisspeptin (*p* < 0.0001, 95% CI: 0.115–0.163, coefficient: 0.154), and its concentration increased as the pregnancy progressed [11]. 

However, the studies conducted on various mammals, including pregnant sheep, cows, pigs, rabbits, horses, rhesus monkeys, and marmoset, showed a low plasma level of kisspeptin and a lack of its increase throughout pregnancy [50]. Interestingly, using a bovine-specific enzyme immunoassay, the continuous increase in plasma kisspeptin concentration during gestation in cows was detected [51]. Based on these results, it seems crucial to use a species-specific enzyme immunoassay or radioimmunoassay as a detection method of kisspeptin. 

Furthermore, kisspeptins are the complex of peptides such as kisspeptin-54, kisspeptin-14, kisspeptin-13, and kisspeptin-10, whereas the studies mentioned above have only focused on detecting one kisspeptin isoform. Another critical issue is the possibility of cross-reaction between the antibody used to measure kisspeptin with other RF-amide-related peptides (RFRP), including prolactin-releasing peptide, RFRP1, RFRP3, neuropeptide FF and AF [52]. Nevertheless, the study using a radioimmunoassay kit with more specific and sensitive antibodies to detect plasma levels of kisspeptin-54, kisspeptin-14, kisspeptin-13, and kisspeptin-10 (rather than only kisspeptin-10), confirmed the correlation of kisspeptin level with gestational age in pregnant women [53]. Sullivan-Pyke et al. obtained the same results and indicated that the kisspeptin-54 level increases during gestation [9].

Considering the reports mentioned above and despite the proven increase in kisspeptin concentration during pregnancy, the levels of kisspeptin mRNA in early and term placenta did not exert statistically significant differences [54]. Moreover, the level of kisspeptin-54 protein, detected by two different methods using western blotting and immunohistochemistry, occurred to be higher in the trophoblast than in the term placenta [28,55]. After analyzing the results of the previous studies, it seems that the kisspeptin expression in the placenta may not necessarily reflect the plasma concentration of this peptide. It could be speculated that the increasing mass of trophoblast cells across gestation might be responsible for the elevated level of kisspeptin in circulation, despite the lowest expression of its protein in the placenta.

## 5. Role of Kisspeptin in Gestational Diabetes Mellitus—A Placenta-Pancreas Crosstalk 

GDM is the most common metabolic disease complicating pregnancies, with an average worldwide prevalence ranging from 5% to 25%, depending on ethnicity, population study, local diagnostic guidelines, maternal age, and accepted screening methods [56]. It is defined as a glucose tolerance disorder that develops or is first recognized during pregnancy [57]. The recommended diagnostic criteria are based on the presence of at least one of the threshold values during the 75 g-oral glucose tolerance test (75g-OGTT): fasting glucose level of 5.1–6.9 mmol/L (92–125 mg/dL) at the first hour ≥ 10.0 mmol/L (≥180 mg/dL) and at the second hour 8.5–11.0 mmol/L (153–199 mg/dL) [58]. Epidemiological studies have revealed that a previous history of impaired glucose tolerance, personal history of medical conditions associated with diabetes (e.g., metabolic syndrome, polycystic ovary syndrome, glucocorticoids use), overweight or obesity, advanced maternal age, dyslipidemia, and history of poor pregnancy outcome or macrosomia in a previous pregnancy seem to be the pivotal risk factors for GDM development [59,60]. It is well established that GDM is associated with a higher risk of numerous maternal and adverse fetal outcomes, including PE, maternal birth trauma, shoulder dystocia, cesarean section, development of type 2 diabetes mellitus (T2DM), cardiovascular complications in a post-partum period in women as well respiratory disorders, hypoglycemia, hyperbilirubinemia in the neonatal period and obesity, metabolic syndrome or impaired glucose homeostasis in the future [61]. 

During pregnancy, the maternal pancreatic β-cells undergo reversible adaptive changes to meet the increased demands for nutrients of the growing fetus [62]. Despite the physiological decline in insulin sensitivity in pregnancy, the functional and structural changes of pancreatic β-cells increase insulin secretion to maintain normoglycemia. The failure of these compensatory mechanisms may result in GDM development [63]. So far, numerous scientific studies have shown that the placenta-derived hormones such as leptin, progesterone, cortisol, estrogen, placental growth hormone, and lactogen are involved in the initiation and progression of insulin resistance [63]. The presence of kisspeptin and KISS-1R in the placenta and pancreatic islet cells has suggested that this peptide may be strongly involved in the pancreas–placenta axis and the development of GDM [19,28]. 

The previous experimental in vitro and in vivo studies revealed that kisspeptin enhanced glucose-stimulated insulin secretion (GSIS) [21,22,64]. The data has shown that kisspeptin increases insulin secretion in human islets in vitro at 17 mM but not at 3 mM glucose concentration [22]. Interestingly, the significant elevation in plasma kisspeptin level in advanced pregnancy with the coexisting peak of insulin resistance remains unclear [49]. However, the corresponding explanation of this phenomenon has been supported by the studies which have indicated that plasma kisspeptin level was negatively correlated with body mass index (BMI), insulin resistance indicator—Homeostatic Model Assessment for Insulin Resistance (HOMA-IR) and serum insulin level [26]. Furthermore, it has been observed that kisspeptin concentration is significantly decreased in other insulin resistance-related conditions such as obesity or polycystic ovary syndrome [26,65,66]. Nevertheless, further studies are necessary to clarify this issue due to the deficiency of studies on the role of kisspeptin in regulating insulin sensitivity, especially in pregnant women. 

The available data that analyzed changes in kisspeptin levels in patients with GDM compared to healthy pregnant women are limited to only a few clinical studies [10,11,64,67,68,69]. It has been reported that women with GDM had significantly lower plasma kisspeptin levels compared to healthy ones [64]. Moreover, the experimental study on the pregnant mice model has revealed that deletion of GPR54 in β-cells resulted in their reduced proliferation capability, which might suggest that kisspeptin plays a physiological role in the regulation of β-cell mass compensation as a response to increased insulin resistance during gestation [64]. Clinical observations have indicated that kisspeptin concentration in GDM women is lower both in the second and third trimester of pregnancy compared to those without GDM (second trimester: 4.51 ± 3.18 (GDM) vs. 10.33 ± 2.65 (controls) nmol/L; third trimester: 11.64 ± 7.65 (GDM) vs. 20.48 ± 7.60 (controls) nmol/L) [68]. These results are contrary to those obtained by Arslan et al., who have shown no differences in the kisspeptin levels in women with GDM and healthy ones [67]. A different research methodology could partially explain distinctions among the obtained results. Kisspeptin concentrations were evaluated in serum, not in plasma, as in other authors’ studies [11,64,68].

A dysregulation in kisspeptin expression has also been detected in the placenta of GDM women [10,69]. KISS-1 mRNA protein was expressed at a higher level in syncytiotrophoblast and cytotrophoblast derived from GDM women than in healthy ones. However, similar relationships have not been found in the case of placental KISS-1R expression [10]. In the light of the above-mentioned results, it seems necessary to clarify the exact mechanisms responsible for the disturbed placenta-kisspeptin-pancreas axis in the course of GDM.

## 6. Role of Kisspeptin in the Pathogenesis of Disorders from the Spectrum of Impaired Implantation and Placentation

As kisspeptin is produced by trophoblast and trophoblast invasion is underway five days after blastocyst transplantation, the careful analysis of its impact on the successful pregnancy and the pathogenesis of its complications from the spectrum of impaired implantation and placentation like miscarriage, PE, and FGR seems to be important [70].

### 6.1. Implantation and Kisspeptin-Induced Miscarriages

Miscarriages are the most common pregnancy complications affecting about 20% of recognized pregnancies [71]. The investigations regarding novel early screening biomarkers to predict the increased risk of pregnancy loss seem crucial. Previous experimental studies have revealed that kisspeptin may regulate embryo implantation by affecting the function of the endometrium and improving the stromal cells’ decidualization [72,73]. The in vivo research conducted on the mice model showed that exogenous supply of kisspeptin leads to amelioration of blastocyst adhesion to collagens via activation of PKC, ERK1/2 pathways, and an increase in MPP-2 and MMP-9 levels as a consequence [74,75]. Furthermore, it is presumed that kisspeptin may increase the level of cytokines that are engaging in the proper implantation, known as leukemia inhibitory factor (LIF). The higher level of LIF is responsible for adequate embryo implantation and stromal decidualization in a mouse model of pregnancy [73,76]. In contrast, it has been demonstrated that kisspeptin agonists may inhibit the invasion and migration of decidual stromal cells by binding with the kisspeptin receptor. It leads to a subsequent decrease in phosphorylation of focal adhesion kinase-steroid receptor coactivator (FAK-Src)/ERK1/2 pathway and MMP-2, MMP-9 protein expressions, which are essential for proper embryo implantation [77].

Interestingly, the engagement of kisspeptin in the modulation of immune tolerance development during gestation has also been examined. One of the proposed mechanisms is the role of kisspeptin in the regulation of natural killer (NK) cell infiltration. It has been observed that the kisspeptin expression in trophoblast cells, derived from pregnancies with recurrent spontaneous abortion, is decreased and correlates with the level of peripheral and decidual NK cells [78]. Moreover, kisspeptin increases the amount of NK CD56^bright^ subtype cells due to TGF-β activation [79]. The studies on the immunomodulatory function of kisspeptin have revealed that it can lead to an increase in the activity of regulatory T-cells (Treg), which produce interleukin 10 (IL-10), the immunosuppressive cytokine [80,81], and an inhibition of T-lymphocyte helpers (Th17) proliferation. It results in a decrease in pro-inflammatory cytokines’ levels, such as interleukin 17A (IL-17A) [82].

The latest clinical observations have confirmed the hypothesis regarding the beneficial effect of kisspeptin on implantation. Kavvasoglu et al. have revealed the lowest plasma concentration of kisspeptin-10 in women with early pregnancy bleeding [83]. The results obtained from a case-control study on the group of 993 asymptomatic pregnant women with a gestation beyond 6 weeks have indicated a decreased serum kisspeptin-54 concentrations in those individuals who were later diagnosed with miscarriage [9]. The researchers indicated that kisspeptin-54 might help discriminate miscarriage from viable intrauterine pregnancy (detection limit of 0.024 ng/mL, intra- and inter coefficients of variation: 5.1% and 8.6%, respectively). Moreover, it has been observed that kisspeptin levels positively correlate with β-chorionic human gonadotropin (β-hCG) and has a comparable diagnostic value (ROC: 0.953 and ROC: 0.994, respectively) [9]. Similar results have been presented by Jayasena et al., who indicated that plasma kisspeptin concentration was 60.4% lower in women with subsequent spontaneous abortion as compared to the control group. It has been found that plasma kisspeptin level also has a higher diagnostic value for miscarriage as compared to serum hCG levels (ROC area under the curve: 0.899 ± 0.025, kisspeptin; 0.775 ± 0.040, hCG). The combined kisspeptin and hCG measurement (OR 0.10; 95% CI 0.06–0.17; *p* < 0.0001) has not shown higher diagnostic accuracy compared to kisspeptin measurement alone [53]. Furthermore, the latest prospective, nested case-control study results are consistent with the previous ones. Its authors underlined that plasma kisspeptin level seems to be a promising biomarker for miscarriage during the first trimester with the area under the receiver-operating characteristic curve of 0.874 (95% confidence interval [CI] 0.844–0.904) [84]. Unlike previous studies, the results of only one research available in the literature are inconclusive [85]. Besides this, it has been revealed that the kisspeptin/GPR54 expression was remarkably lower at the maternal-fetal interface in women with recurrent spontaneous abortion compared to controls. These observations have also been confirmed in an experimental mice model [86].

The studies on the trophoblast and decidua cells derived from women with recurrent spontaneous abortion revealed the decreased expression of the GPR54 receptor [29,87]. Moreover, the studies in women with unexplained infertility who underwent assisted reproductive procedures indicated that the lower kisspeptin concentration was associated with a minor capability of embryo implantation. [88,89,90]. The above data suggests a vital kisspeptin role in embryo implantation and the development of immune tolerance. Its reduced level could be associated with a greater risk of pregnancy loss.

### 6.2. Kisspeptin in Placentation and Pathophysiology of Placental Diseases

The process of placentation begins on the 7th day after fertilization. The trophoblast invasion resembles the process of cancer invasion because, during its incorporation into decidua, the increased proliferation, invasion capabilities, reduced apoptosis, and enhanced blood supply are observed [91]. The detection of kisspeptin/KISS-1R expression in human trophoblast at a higher level during the early stage of gestation than in the term placenta suggested the potential significance of kisspeptin in the regulation of invasive and migratory properties of trophoblast cells to ensure the sufficient placentation [54].

The first evidence confirming the role of kisspeptin-54 in the regulation of invasion processes by MMPs, a family of Zn- and Ca^2+^—dependent endopeptidases, has been presented by Yoshioka et al. on the in vitro model of renal carcinoma cells [92]. This observation has been confirmed by other studies, which strongly indicated that kisspeptin decreased trophoblast cells’ invasion by attenuation of MMPs expression dose-dependent [9,28,93]. Francis et al. demonstrated that kisspeptin-10 treatment reduced the expression of MMP-2 in the primary trophoblast cells and subsequently disturbed their migratory capability [46]. Moreover, kisspeptin mediates a decrease in MMP-1, 3, 7, 10, 14, VEGF, and increased TIMP-1, 3 levels by affecting the ERK1/2 signaling pathway in primary trophoblast cells [46]. The research conducted on various tumor cultured cells has indicated that kisspeptin modulates MMP-2, 9 expressions via blocking translation of nuclear factor κB (NF-κB) which led to their impaired invasiveness [94,95].

The latest in vitro experimental study has shown that TGF-β, which is also expressed in the human placenta and is responsible for the regulation of cellular processes, seems to inhibit human trophoblast cells’ invasion by upregulating kisspeptin expression through the ERK1/2 signaling pathway [48]. Furthermore, numerous studies have revealed that kisspeptin may restrain the trophoblast growth by activating apoptosis in a dose-depended manner, together with pro-inflammatory cytokines, including tumor necrosis factor α (TNF-α) [96,97,98].

It is well-established that the regulation of neovascularization and angiogenesis plays a pivotal role in placentation, and its disturbances could finally result in the development of different placenta-related gestational complications such as PE and FGR [99]. So far, VEGF has been proposed as one of the essential pro-angiogenic factors [99,100,101]. Kisspeptin was noticed to inhibit VEGF expression and thus control the formation of embryonic vessels and the growth of new ones in the already existing vascular system [46]. Sharkey et al. have observed that the plasma concentrations of VEGF in physiological pregnancy were significantly lower than in pregnancies complicated by PE (11.7 ng/mL vs. 32.7 ng/mL, respectively) [102]. PE is believed to be a consequence of the decreased placental perfusion caused by the lack of transformation of the spiral arteries into uteroplacental arteries [103]. 

Kisspeptin has been proposed to be a regulator of vascular tone by deregulation of eicosanoid synthesis that may impair the function of the maternal cardiovascular system and promote the development of hypertension [104]. Previous studies have confirmed that KISS-1R expression was detected in maternal smooth muscles, aorta surface, coronary arteries, and umbilical cord [104,105]. Moreover, it has been observed that kisspeptin-10 exhibits the angiogenesis-inhibiting and vasoconstriction effects that confirms its pivotal role in the pathogenesis of placenta-related complications [106]. These observations regarding the role of kisspeptin in the pathogenesis of impaired placentation make it an essential factor in developing placenta-related diseases, including PE and FGR.

#### 6.2.1. Kisspeptin and Preeclampsia

PE constitutes one of the leading causes of maternal and perinatal mortality worldwide. It is estimated to complicate 2–8% of pregnancies globally [107]. PE is characterized by a gestational disorder associated with de-novo development of hypertension after 20. hbd accompanied by new-onset proteinuria or organ dysfunction [107]. Although hypertension and proteinuria are considered the classical criteria to diagnose PE, new-onset of other symptoms are recommended for PE diagnosis, including thrombocytopenia, renal insufficiency, impaired liver function, pulmonary edema, or headache unresponsive to medication [107]. PE can be divided into the early- and late-onset subtypes that are diagnosed before or from 34. hbd, respectively. Early-onset phenotype is associated with impaired placental development and subsequent FGR, while the late-onset one may be related mainly to maternal endothelial dysfunction [108]. One of the most important factors engaged in the pathogenesis of PE is abnormal placental function. It has been demonstrated that the placenta from pregnancies diagnosed with PE is characterized by poor differentiation and immaturity [109]. Moreover, placental vascular dysfunction, including incomplete spiral artery remodeling and endothelial damage, have been proposed as crucial abnormalities responsible for PE development [110]. 

Numerous clinical studies have revealed that preeclamptic women presented elevated plasma [11] or serum [48,111] kisspeptin levels compared with healthy individuals. The research conducted by Abbara et al. has shown that plasma kisspeptin level in pregnancies complicated by hypertensive disorders was significantly increased than in healthy ones (OR of hypertensive pregnancies was increased by 30% (95% CI, 16–47%; *p* < 0.0001) for every 1 nmol/L increase in kisspeptin concentration) [11]. However, the previous studies have presented lower kisspeptin plasma [68,112,113,114] or serum [115,116,117] levels in PE women. Different detection methods may partially explain these divergencies because some authors have measured total kisspeptin concentrations, while others have only one selected peptide. Furthermore, it has been observed that KISS-1 mRNA expression seems to be increased in the PE placenta [116,118,119,120]. The differences may also be associated with the fact that circulating kisspeptin levels increased consistently across gestation, whereas kisspeptin expression in the placenta has been found to increase with the peak in the first trimester and subsequently decline [28,55]. Cartwright et al. have revealed that the lower protein and mRNA expression of kisspeptin with increased KISS-1R expression in the PE placenta may partially explain the lower plasma or serum levels of kisspeptin [55]. Moreover, the authors have suggested that increased activity of KISS-1R can result in trophoblast immaturity and PE development as a consequence [55]. 

Interestingly, plasma kisspeptin-10 and kisspeptin-54 concentrations have been found to correlate with the severity of PE [113,114]. The Kisspeptin-10 level was significantly decreased in women with the severe form of PE, and its level was remarkably lower in severe PE in the second trimester than in the third one (1.59 ± 0.26 vs. 2.39 ± 0.57; *p* < 0.0001) [114]. Furthermore, Adali et al. have noticed that plasma kisspeptin-54 negatively correlated with daily proteinuria (r = −0.299; *p* < 0.01) and with mean arterial pressure (r = −0.316, *p* < 0.01) [113]. The authors have also revealed significant differences in kisspeptin-54 concentrations in women with PE with abnormal Doppler velocimetry compared to those with normal ones (0.84 ± 0.30 vs. 2.08 ± 0.29, respectively; *p* = 0.018) [113].

It has been observed that plasma kisspeptin level is increased in women who experienced late-onset PE compared to gestational-age matched healthy pregnancies (68.7 ± 93.4 pg/mL vs. 68.5 ± 57.9 pg/mL; *p* = 0.004). The optimal cut-off of kisspeptin was 67.2 pg/mL with 53% sensitivity, 76% specificity, and diagnostic accuracy of 64% [121]. These results may partially explain the role of kisspeptin in vascular tone regulation, which is also considered in the pathogenesis of late-onset PE. Qiao et al. have revealed the elevated KISS-1 mRNA expression in the placenta-derived from women with early-onset PE (2.2-1.8-fold upregulation vs. controls) [118]. Because kisspeptin inhibits trophoblast migration and invasion, it can be postulated that increased KISS-1 expression in the placenta may contribute to early-onset PE development. The summary of the available research regarding the changes in kisspeptins concentration in different sample types and KISS-1 mRNA and KISS-1R expressions in the placenta of pregnancies complicated by PE was presented in Table 1. 

#### 6.2.2. Kisspeptin and Fetal Growth Restriction

It has been widely accepted that birth weight plays a pivotal role in human health. Fetal growth restriction (FGR), also previously known as intrauterine growth restriction (IUGR), is one of the most significant causes of perinatal morbidity and mortality [122]. Epidemiological studies have indicated that FGR, together with the cases of small for gestational age (SGA) fetuses, are responsible for nearly 30% of unexplained stillbirth in the late third trimester [123]. FGR is defined as the failure of the fetus to achieve the programmed birth weight after excluding internal factors, whereas the term SGA in most of the guidelines refers to the fetuses with estimated weight on ultrasound between the 3rd and 10th percentile for gestational age without signs of growth failure or newborns with a birth weight below the 10th percentile [122,124]. Various maternal and environmental factors have already been proposed to be involved in the pathogenesis of FGR. The impaired uterine–placental perfusion seems to be the leading cause of FGR development [122,125]. Currently, no intrauterine therapeutic strategies are available for FGR treatment; therefore, searching for FGR predictive biomarkers seems to be crucial [126].

In recent years, much attention has been paid to the potential role of kisspeptin in regulating intrauterine fetal development. Numerous studies have strongly linked the decreased kisspeptin level and the low birth weight in women with uncomplicated pregnancies [127,128]. However, Comert et al. have shown that maternal kisspeptin levels during the first trimester did not differ significantly between newborns classified into three groups according to birth weight percentiles (≤10%—SGA; 10–90%—appropriate for gestational age; >90%—large for gestational age), [129]. Simultaneously, Cetković et al. have obtained no distinctions in placental mass, newborn weight at delivery, and maternal kisspeptin-54, kisspeptin-14, and kisspeptin-10 levels in women with PE and healthy ones [68].

The previous clinical observations have indicated that the lower kisspeptin-10 concentration in women diagnosed with early pregnancy bleeding is associated with a subsequent increased risk of FGR development by 10% [83]. Moreover, it has been noticed that there is a positive correlation between kisspeptin-10 level in the third trimester and estimated fetal weight (r = 0.395, *p* = 0.012) in women with uncomplicated pregnancies and in the second (r = 0.760, *p* = 0.001), third (r = 0.920, *p* = 0.0001) trimesters with severe PE [114]. Other retrospective and nested case-control studies have revealed decreased maternal kisspeptin levels in pregnancies complicated by FGR compared to healthy women, consistent with the previous data [11,117]. Furthermore, the latest study has shown that kisspeptin level is lower in the late first and third trimester (*p* = 0.040; *p* = 0.025, respectively) in women diagnosed with FGR than in control pregnancies, which may suggest when it should be measured [11]. Interestingly, the increase in kisspeptin level throughout gestation was significantly lower in pregnancies affected by FGR than in uncomplicated ones (*p* = 0.004). The odds ratio of FGR adjusted for maternal age, ethnicity, BMI, smoking status, and parity were decreased by 28% (95% CI, 4–46%) for every 1 nmol/L increase in plasma kisspeptin (*p* = 0.025). Hence this peptide may be proposed as a promising biomarker of FGR [11]. All of the available studies to prove the role of kisspeptins and KISS-1R in FGR are presented in Table 1. 

## 7. Kisspeptin and Neonatal Outcomes

Numerous studies have strongly indicated that preterm birth affects approximately 11% of births worldwide, and it plays a pivotal role in the development of different adverse neonatal outcomes, including higher risk of neonatal mortality, respiratory distress syndrome, necrotizing enterocolitis, intraventricular hemorrhage or future metabolic disturbances, and cerebral palsy [130]. Oxytocin is one of the most crucial hormones in parturition, mainly due to inducing uterine contraction [131]. It is worth underlying that endometrial inflammation, reflected by an increase in inflammatory markers including endometrial cytokines and angiogenetic factors, has been found to play a pivotal role in the pathogenesis of preterm delivery [132]. However, some authors have indicated that oxytocin exerts different effects after degradation to active fragments, and its anti-inflammatory properties are postulated [133]. 

The previous study on the animal model has indicated that intracerebroventricular injection of kisspeptin-10 increased the rate of activated oxytocin neurons only on 18–21 days of pregnancy. It should be underlined that hypothalamic KISS-1R did not change during gestation [134]. Moreover, it has been reported that the placental expression of kisspeptin was significantly higher in women who experienced preterm vaginal delivery than in those with term vaginal delivery. However, maternal plasma kisspeptin concentrations were comparable in these groups of women [135]. In contrast, Abbara et al. observed higher plasma kisspeptin levels in pregnancies affected by preterm birth (*p* = 0.014). 

## 8. Conclusions and Future Perspectives

**Figure 2:**
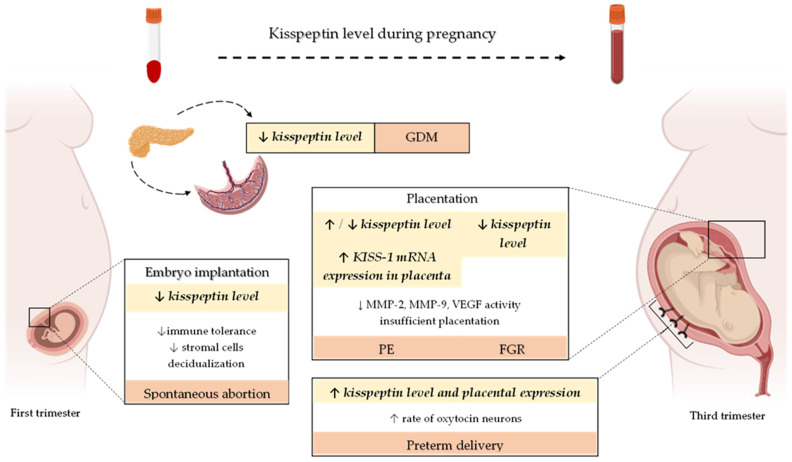
Changes in plasma/serum kisspeptin level and its expression in the human placenta during the physiological course of gestation as well as the most common pregnancy-related complications. Abbreviations: *KISS-1*—kisspeptin; GDM—gestational diabetes mellitus; PE—preeclampsia; FGR—fetal growth restriction; ↓—decreased; ↑—increased.

Kisspeptin exerts the biological action by binding with its receptor KISS-1R, mainly expressed in the human placenta, umbilical cord, and maternal vascular smooth muscle cells. This comprehensive review summarizes the available evidence indicating a pleiotropic role of kisspeptin in embryo implantation, adequate placentation, and the pathogenesis of the most common gestational complications (Figure 2). Based on the research results regarding kisspeptin in pregnancy, the placenta is postulated as its primary source. Available data suggest that disturbances in kisspeptin concentrations may be associated with several pregnancy complications, including miscarriage and the development of GDM, PE, and FGR. Hence, its role as an early predictor of these complications might be considered. However, the research results are inconclusive, and many issues, such as the type of samples and processing methods, have still to be elucidated and standardized. Moreover, extensive prospective cohort studies should be performed to accurately determine kisspeptins’ sensitivity and specificity in predicting pregnancy-related complications. Defining the gestation period when kisspeptin determination would be necessary for predicting pregnancy disorders is also essential. 

Despite the thorough understanding of the kisspeptin/KISS-1R complex interaction pathways, little is known about the factors that regulate its functions and activity. Therefore, further studies on the role of kisspeptin in pregnancy-related complications and its signaling are necessary.

**Table 1:** Summaries of the studies conducted on the role of kisspeptin in placental diseases.

Disorder	KISS Derivatives	Ethnicity (Country)	Sample Type	Trimester of Pregnancy	Model (Number) of the Study Groups	Description of Findings	Authors
	KISS	Mixed (White, Black, Asian, Other)	plasma	All trimester	PE (20): mild (7), severe (13) PIH (12) CG (265)	↑ KISS level in HDP women vs. CG The rate of rise in KISS with gestation ↑ in pregnancies affected by HDP than in CG OR of HDP increased by 30% (95% CI, 16–47%; *p* < 0.0001) for every 1 nmol/L increase in plasma kisspeptin	Abbara et al., 2022 [11]
	KISS-1	Asian (Turkey)	plasma	<34. hbd ≥34. hbd	early-onset PE (20) vs. CG (20) late-onset PE (45) vs. CG (40)	No difference ↑ level of KISS-1 in late-onset PE vs. CG	Ibanoglu et al., 2022 [121]
	KISS-1	Asian (China)	serum	Second Third	PE women (25) CG (25)	↑ levels of KISS-1 and TGF-β1 in PE women vs. CG	Fang et al., 2022 [48]
	KISS-1	Asian (China)	human trophoblast cell line placenta serum	Second Third	PE women (17) CG (16)	EGF downregulated KISS-1 by activating EGFR-mediated PI3K/AKT signaling pathway ↓ EGF and ↑ KISS-1 level in PE women vs. CG	Fang et al., 2021 [111]
	KISS-10	Asian (Jordan)	plasma	Second Third	PE women (60) CG (40)	↓ KISS-10 level in PE women vs. CG KISS-10 levels correlate positively with β-hCG and negatively with LH, and FSH in PE women in the third trimester	Al-Kaabi et al., 2020 [112]
	KISS-10	Asian (Iraq)	plasma	Second Third	PE women (60) Mild form (39) Severe form (21) CG (40)	↓ KISS-10 level in PE vs. CG ↓ KISS-10 level in severe PE vs. mild one ↓ KISS-10 level in severe PE during the second trimester vs. the third one	Ziyaraa et al., 2016 [114]
	KISS-10 GPR-54	European (UK)	placenta serum (maternal, umbilical cord blood	Third	PE women (19) CG (30)	↑ KISS expression in PE placenta vs. CG No differences in KISS-1 and GPR-54 mRNA expressions in placentas ↓ KISS-10 level in serum in SG vs. CG No significant distinctions in KISS-10 levels in cord blood between SG and CG	Matjila et al., 2016 [116]
	KISS-1KISS-1R	European (UK)	placenta	First Third	First trimester (10) PE women at delivery (10) CG (10)	↓ KISS-1 and KISS-1R expression between early and term pregnancy PE vs. normal pregnancy placental samples: ↓ KISS-1 expression and ↑ expression of KISS-1R	Cartwright et al., 2012 [55]
	KISS	European (UK)	plasma	16. hbd 28. hbd 36. hbd	Obese women with PE (11) Uncomplicated pregnancy in obese women (158) Lean pregnant women—CG (48)	Maternal KISS levels ↑ during pregnancy ↓ KISS level in obese women with PE at 16. hbd compared to obese pregnant women without PE and CG Optimal cut-off concentration of KISS at 16. hbd to predict PE—596 pmol/L (sensitivity: 85.7%; specificity: 71.4%)	Logie et al., 2012 [127]
	KISS-1 GPR-54	Asian (China)	placenta	23^+0^–33^+6^. hbd 34^+0^–39^+0^. hbd	early-onset PE (36) vs. CG (40) late-onset PE (40) vs. CG (40)	↑ KISS-1 mRNA expression in early-onset SG vs. CG No differences in KISS-1 mRNA expression in late-onset PE No differences in GPR-54 expression	Qiao et al., 2012 [118]
	KISS-54	Asian (Turkey)	serum	First	Women who developed PE (31) CG (30)	↓ KISS-54 level in SG vs. CG (AUC: 0.797 to predict PE)	Madazli et al., 2012 [115]
	KISS-54	Asian (Turkey)	plasma	Third	Mild PE (15) Severe PE (24) CG (50)	↓ KISS-54 level in PE vs. CG No differences between mild and severe PE	Adali et al., 2012 [113]
	KISS-54 KISS-14 KISS-10	European (Serbia)	plasma	Second Third	PE (28) GH (18) CG (25)	↓ KISS-54 level in PE vs. CG No differences in KISS level between GH and CG	Cetković et al., 2012 [68]
	KISS-1	Asian (China)	placenta human trophoblast cell line	Third	PE women (47) CG (30) Human trophoblast cell line transfected (SG) and non-transfected one (CG) with KISS-1 vector	↑ KISS-1 mRNA and protein expression in PE women vs. CG ↓ MMP-9, MMP-2 mRNA, and protein expression in PE vs. CG No differences in cell proliferation between SG and CG ↓ invasion ability in SG compared to CG	Zhang et al., 2011 [119]
	KISS-1	Amerindian (Mexico)	placenta	Third	PE women (27) CG (27)	↑ KISS-1 expression in SG vs. CG	Vazquez-Alaniz et al., 2011 [120]
	KISS-54	European (UK)	plasma	Third	PE women (8) PIH (19) CG (78)	No differences	Nijher et al., 2010 [136]
	KISS-54	European (UK)	serum	Second	PE women (57) CG (317)	↓ KISS-54 level in PE individuals vs. CG	Armstrong et al., 2009 [117]
	KISS-1	European (Italy)	whole blood	Third	PE (6) CG (30)	↓ KISS-1 mRNA expression in PE vs. CG	Farina et al., 2006 [137]
Fetal Growth Restriction	KISS	Mixed (White, Black, Asian, Other)	plasma	All trimester	FGR or SGA (17) CG (265)	↓ KISS level in FGR vs. CG (especially in the late first and third trimester) Lower increase in KISS level during gestation in FGR vs. CG KISS in diagnosis FGR: OR 0.72 (0.54–0.96), *p* = 0.025	Abbara et al., 2022 [11]
	KISS-10	Asian (Iraq)	plasma	Second Third	PE women (60) Mild form (39) Severe form (21) CG (40)	Positive correlation between KISS level in the third trimester and EFW (r = 0.395, *p* = 0.012) in CG, and in the second (r = 0.760, *p* = 0.001), third (r = 0.920, *p* = 0.0001) trimesters in severe PE Negative correlation between KISS level in the third trimester and FBW in CG (r = −0.410, *p* = 0.009)	Ziyaraa et al., 2016 [114]
	KISS-1	Mixed (Australia)	Whole blood (maternal)	26–30 hbd	Late-onset FGR (40) CG (80)	↑ KISS-1 expression in FGR vs. CG AUC for KISS01 as a discriminative marker for FGR: 0.64, *p* = 0.01 (FGR: 0.19 (0.08–0.81) vs. CG: 0.96 (0.13–2.85)	Whitehead et al., 2016 [138]
	KISS-1 KISS-1R	Animal model (rat)	Placenta	16. and 22. day of gestation	Dexamethasone-induced FGR (6) CG (6)	↑ KISS-1 mRNA expression in FGR vs. CG ↑ KISS-1R mRNA expression in 16. day of gestation and ↓ in 22. day of gestation in FGR vs. CG	Mark et al., 2013 [139]
	KISS-54 KISS-14 KISS-10	European (Serbia)	plasma	Second Third	PE (28) GH (18) CG (25)	No correlation between KISS-54, placental, and birth weight at delivery in all groups	Cetković et al., 2012 [68]
	KISS	European (UK)	plasma	16. hbd 28. hbd 36. hbd	Obese women with PE (11) Uncomplicated pregnancy in obese women (158) Lean pregnant women—CG (48)	Lower KISS levels at 16. hbd were associated with lower birth weight (r = 0.16, *p* = 0.06)	Logie et al., 2012 [127]
	KISS-1	Mixed (Australia)	Whole blood Placenta	Third (<34. hbd)	FGR (20) Preterm Birth (15—blood samples, 8—placenta) Term Birth (8—placenta)	↑ KISS-1 RNA expression in maternal blood in FGR vs. women with preterm delivery ↑ KISS-1 RNA expression in the placenta in FGR vs. women with preterm and term delivery	Whitehead et al., 2012 [140]
	KISS-10	Asian (Turkey)	Plasma	First	Women with early pregnancy bleeding (20) CG (20)	↓ KISS-10 level in the first trimester in SG and was associated with an increased risk of IUGR (10% vs. 0%)	Kavvasoglu et al., 2012 [83]
	KISS-54	European (UK)	Serum	Second	FGR (118) CG (317)	↓ KISS-54 level in FGR vs. CG	Armstrong et al., 2009 [117]
	KISS-54	European (Netherlands)	Plasma	8–14 hbd.	SGA (31) CG (31)	↓ KISS-54 level in SGA vs. CG	Smets et al., 2008 [128]

↓—decreased; ↑—increased; CG—control group; SG—study group; PE—preeclampsia; KISS-10—kisspeptin-10; KISS-54—kisspeptin-54; KISS-1R—kisspeptin 1 receptor; LH—luteinizing hormone; FSH—follicle-stimulating hormone; β-hCG—human chorionic gonadotropin; PIH—pregnancy-induced hypertension; GH—gestational hypertension; MMP-9—matrix metalloproteinase 9; MMP-2—matrix metalloproteinase 2; EGF—epidermal growth factor; EGFR—epidermal growth factor receptor; GPR-54—G-protein coupled receptor for kisspeptin-54; MAP—mean arterial pressure; HDP—hypertensive disorders of pregnancy; FGR—fetal growth restriction; SGA—small for gestation age; AUC—area under the curve; OR—odds ratio; EFW—estimated fetal weight; FBW—fetal birth weight.

## Data Availability

The data used to support the findings of this study are included within the article.
